# Dietary inflammatory index (DII) is correlated with the incidence of non-alcoholic fatty liver disease (NAFLD): Fasa PERSIAN cohort study

**DOI:** 10.1186/s40795-023-00738-5

**Published:** 2023-07-11

**Authors:** Adib Valibeygi, Ali Davoodi, Azizallah Dehghan, Farhad Vahid, James R. Hébert, Mojtaba Farjam, Reza Homayounfar

**Affiliations:** 1 Student Research Committee, Fasa University of Medical Sciences, Fasa, Iran; 2 Noncommunicable Diseases Research Center, Fasa University of Medical Sciences, Fasa, Iran; 3 Department of Precision Health, Nutrition and Health Research Group, Luxembourg Institute of Health, Strassen, Luxembourg; 4 Department of Epidemiology and Biostatistics, Arnold School of Public Health, University of South Carolina, Columbia, SC USA; 5 South Carolina Statewide Cancer Prevention and Control Program, University of South Carolina, Columbia, SC USA; 6 National Nutrition and Food Technology Research Institute, Faculty of Nutrition and Food Technology, Shahid Beheshti University of Medical Sciences, Tehran, Iran

**Keywords:** Dietary inflammatory index, Non-alcoholic fatty liver disease, Fatty liver index, Diet, Hypertriglyceridemia, Hypercholesterolemia

## Abstract

**Background:**

Non-alcoholic fatty liver disease (NAFLD) is a prevalent liver disease predisposing patients to life-threatening conditions, including cirrhosis. There is evidence that the incidence of NAFLD is related to the individuals’ dietary patterns; however, it is still remaining unknown whether the inflammatory potential of various foods/dietary patterns can directly predict a higher incidence of NAFLD.

**Methods:**

In this cross-sectional cohort study, we investigated the relationship between the inflammatory potential of various food items and the incidence/odds of NAFLD. We used data from Fasa PERSIAN Cohort Study comprising 10,035 individuals. To measure the inflammatory potential of diet, we used the dietary inflammatory index (DII®). Fatty liver index (FLI) was also calculated for each individual to identify the presence of NAFLD (cut-off = 60).

**Results:**

Our findings showed that higher DII is significantly associated with increased incidence/odds of NAFLD (OR = 1.254, 95% CI: 1.178—1.334). Additionally, we found out that higher age, female gender, diabetes mellitus, hypertriglyceridemia, hypercholesterolemia, and hypertension are other predictors of developing NAFLD.

**Conclusions:**

It can be concluded that consuming foods with a higher inflammatory potential is associated with a greater risk of developing NAFLD. Additionally, metabolic diseases, including dyslipidemia, diabetes mellitus, and hypertension, can also predict the incidence of NAFLD.

## Introduction

Non-alcoholic fatty liver disease (NAFLD) is defined as an accumulation of excessive fat in the liver over 5% of the liver weight, mainly in the form of triglycerides, in individuals not taking more than 10 grams of alcohol daily [1]. NAFLD is a prevalent disease and a major risk factor for serious conditions, including hepatitis, hepatocellular carcinoma, and metabolic syndrome [2–4]. According to previous investigations, about one-fourth of the adult population worldwide suffer from NAFLD [5]. Studies in Iran show that the prevalence of NAFLD in the adult population ranges from 21.5% to 43.8% in various geographical regions [6–8].

Considering the high prevalence and potential consequences of the disease, many investigations have been undertaken to identify predictors of NAFLD. Existing evidence supports the association between dietary patterns and the risk of developing NAFLD. Studies that have assessed foods' macro-and micronutrients and their metabolic effects reveal that consumption of red meat, eggs, high-fat dairy products, fried potatoes, and diets rich in omega 6 and simple carbohydrates are associated with an increased risk of NAFLD [9, 10]. By contrast, fish consumption, low-fat dairy products, natural fresh fruits and vegetables, whole-grain cereals, and dietary patterns rich in anti-oxidants and vitamins (e.g., Mediterranean diet) are associated with decreased risk of NAFLD [11–13].

It is hypothesized that the effect of different foods and dietary patterns on the risk of NAFLD is mediated by pro-and anti-inflammatory markers (e.g., TNF-α and IL-6) [14–16]. Additionally, the IL-6 level is found to be correlated with the severity of the disease [14]. Therefore, it stands to reason that the inflammatory potential of different foods and dietary patterns might influence the chance of developing NAFLD. However, little evidence directly addresses the association between the inflammatory properties of foods and the incidence of NAFLD. Therefore, the present study is designed to evaluate the association between diet-associated inflammatory potential using the Dietary Inflammatory Index (DII^®^) and NAFLD in the participants of the Fasa PERSIAN cohort study. There is a previous case-control study carried out in Iran, which showed that consumption of pro-inflammatory foods is associated with increased risk of NAFLD [17]. Howver, this study had much smaller sample size (295 cases and 704 controls), while our study has the sample size of nearly 10,000 individuals. We hypothesize that the DII score is significantly and positively associated with NAFLD incidence, indicating that consuming foods with a higher DII score is associated with an increased risk of NAFLD.

## Material and method

### Population

The present cross-sectional study used data extracted from the Fasa PERSIAN cohort study, a longitudinal population-based study conducted from November 2014 to June 2019. This study's sample population comprises 10,035 individuals aged 35-70 who were not disabled physically or mentally and were selected randomly from the people of the Sheshdeh rural area (with 41000 inhabitants) who had been living there for at least 9 months. This cohort study was designed to evaluate the risk factors of non-communicable diseases in this population. In this study, trained rural healthcare workers, called Behvarzes, were recruited to interview the participants and collect the necessary data and samples [18, 19].

**Fig. 1:**
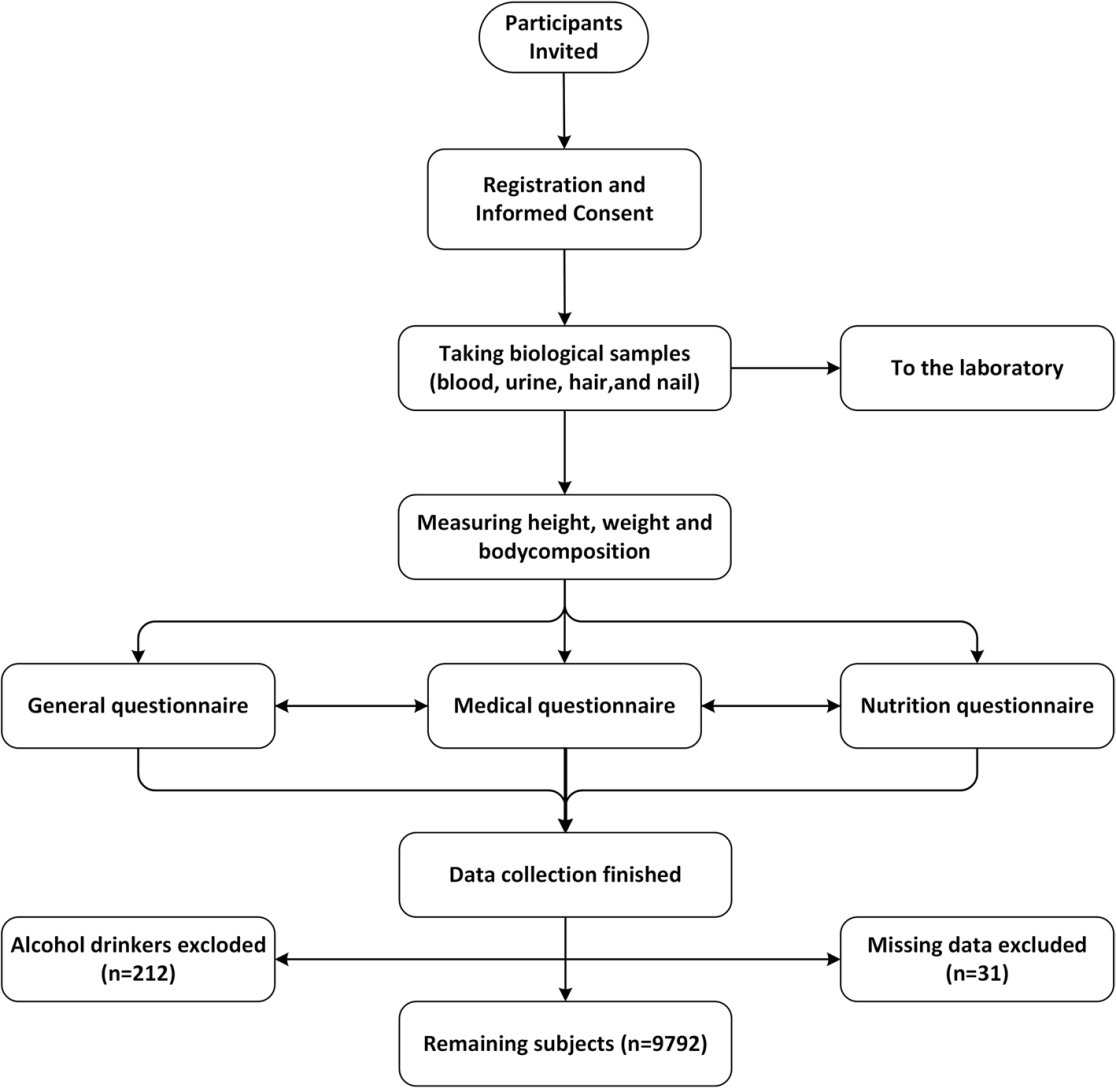
Flowchart of the registration and data collection process, Fasa PERSIAN cohort study, 2014–2019. (Made by Microsoft Visio)

After the primary registration, the biological samples were taken from the participants. Then, a precise physical examination was performed, and anthropometric variables were measured. Finally, a detailed history including demographic, socioeconomic, habitual, nutritional, and medical information was obtained from the participants through the interview. Since our study determines the incidence of NAFLD, alcohol drinkers (*n *= 243) were excluded [18]. The flowchart of the study process is depicted in Fig. 1.

### Assessment of dietary intakes

To assess the nutritional status of each individual, a 125-item FFQ has been used to evaluate their dietary habits and intakes over the past year. A trained nutritionist asked the participants to report the amount, frequency, and portion size of each food item they have been consuming over the recent 12 months (on a daily, weekly, monthly, and annual basis). Regarding the differences in foods and eating habits in different cultures, necessary changes were made to this questionnaire based on Iranian foods and dietary patterns [20]. We used Nutritionist IV software (version 7.0) [17] to determine each food's nutritional and energy content.

### Assessment of DII

DII, designed in 2009 and revised in 2014, is a valid index for measuring various foods' pro- or anti-inflammatory potentials [21]. This index is based on the effect of diet on the serum level of six different inflammatory markers (i.e., IL-4, IL-6, IL-10, TNF-α, IL-1β, and C-reactive protein) and has been validated in over 40 studies around the world against these and other biomarkers [22]. The DII has been used previously in over 750 studies and has been demonstrated to be a precise, valid, and reliable tool for evaluating the inflammatory properties of diets [20, 23, 24].

### Assessment of FLI

FLI: FLI is a simple tool used to diagnose fatty liver disease. To calculate the FLI, we used the following formula designed by Bedogni et al. in 2006 [25].

$$\mathrm{FLI}=(e(0.953\times \mathrm{ln}(\mathrm{TG})+0.139\times \mathrm{BMI}+0.718\times \mathrm{ln}(\mathrm{GGT})+0.053\times \mathrm{WC}-15.745))/(1+\mathrm{e}(0.953\times \mathrm{l n}(\mathrm{TG})+0.139\times \mathrm{BMI}+0.718\times \mathrm{ln}(\mathrm{GGT})+0.053\times \mathrm{WC}-15.745)\times 100$$

The variables of this algorithm include serum triglycerides (TG)(mg/dl), gamma-glutamyl-transferase (GGT)(U/L), BMI, and waist circumference (WC)(cm). FLI ranges from 0 to 100, with an FLI of less than 30 ruling out and an FLI of 60 or more ruling in fatty liver disease. This algorithm was found to have an accuracy of 84% (95% CI 0.81–0.87) in diagnosing fatty liver disease. The negative and positive likelihood ratios were measured at 0.2 and 4.3, respectively [25]. The validity and reliability of this measure are confirmed by multiple population-based investigations [1, 26, 27].

#### Statistical analysis

SPSS version 24 was used for data analysis. We reported quantitative variables as mean ± standard deviation (SD) and qualitative variables as frequencies. Independent samples T-test and Chi-square test were used for comparative analysis of quantitative and qualitative data. Finally, logistic regression and ordinal regression analysis were performed, and the level of significance, odds ratio (OR), and 95% CI were reported. *P *<0.05 was considered to be statistically significant. The regression analyses were adjusted for confounding variables influencing the relationship between DII and NAFLD, including diabetes mellitus, hypertriglyceridemia, hypercholesterolemia, body mass index (BMI), energy intake, and physical activity.

#### Ethical approval

The study procedure was utterly consistent with the Helsinki declaration. All subjects were informed about the study procedure and their rights, and an informed consent form was obtained. The participants could leave the study as they wished. The study also was approved by the institutional review board (IR.FUMS.REC.1399.138).

## Results

A total of 10,035 individuals were included in this study, of whom 212 (2.11%) were excluded due to alcohol consumption and 31 subjects (0.3%) were excluded due to missing data. Therefore, the study evaluated 9792 subjects (43.9% male). The distribution and percentages of quantitative and qualitative variables in the total population and also among genders are shown in Table 1.

**Table 1:** Distribution of qualitative and quantitative variables among the 9792 male and female subjects, Fasa PERSIAN cohort study, 2014–2019

Variable	Total	Female	Male	*P*-value
Age (years)	48.74 ± 9.56	48.64 ± 9.53	48.86 ± 9.61	0.26
DM	12.4%	16%	7.9%	< 0.0001*
HTN	20.3%	27.2%	11.6%	< 0.0001*
DBP (mmHg)	74.71 ± 11.94	74.90 ± 12.05	74.47 ± 11.79	0.07
SBP (mmHg)	111.49 ± 18.46	112.09 ± 19.15	110.72 ± 17.51	< 0.0001*
MAP (mmHg)	86.96 ± 13.42	87.29 ± 13.74	86.55 ± 13.00	0.006*
BMI (Kg/m^2^)	25.69 ± 4.82	26.85 ± 4.81	24.21 ± 4.41	< 0.0001*
MET (ml/kg/min)	41.41 ± 11.25	38.43 ± 6.72	45.22 ± 14.31	< 0.0001*
WBC (count/µl)	6.36 ± 1.34	6.32 ± 1.32	6.41 ± 1.36	0.002*
FBS (mg/dl)	92.70 ± 29.59	94.56 ± 33.05	90.33 ± 24.28	< 0.0001*
Triglyceride (mg/dl)	131.57 ± 82.19	128.31 ± 74.23	135.73 ± 91.18	< 0.0001*
Cholesterol (mg/dl)	185.28 ± 39.13	190.28 ± 39.44	178.90 ± 37.78	< 0.0001*
AlkP (IU/l)	209.53 ± 71.65	208.24 ± 75.61	211.17 ± 66.23	0.04*
HDL (mg/dl)	51.20 ± 15.96	54.17 ± 16.41	47.40 ± 14.51	< 0.0001*
LDL (mg/dl)	107.74 ± 32.82	110.41 ± 33.56	104.33 ± 31.53	< 0.0001*
GGT (IU/l)	22.80 ± 21.45	20.43 ± 20.06	25.81 ± 22.75	< 0.0001*
Energy intake (kcal/day)	2929.9 ± 1148.2	2819.9 ± 1113.5	3070.4 ± 1176.39	< 0.0001*
FLI	39.73 ± 27.72	43.49 ± 27.62	34.94 ± 27.11	< 0.0001*
DII	-0.30 ± 2.05	-0.26 ± 2.21	-0.35 ± 1.83	0.03*
HT	28.1%	27.4%	29.1%	0.06
HC	32.3%	36.6%	26.8%	< 0.0001*
Opium consumption	22%	1.8%	47.7%	< 0.0001*
Smoking	25.9%	5%	52.5%	< 0.0001*
NAFLD	26.9%	30.9%	21.8%	< 0.0001*
Total count	9792	5494	4298	-

*DM* diabetes mellitus, *HTN* hypertension, *NAFLD* non-alcoholic fatty liver disease, *DBP* diastolic blood pressure, *SBP* systolic blood pressure, *MAP* mean arterial pressure, *BMI* body mass index, *MET* metabolic equivalent, *WBC* white blood cell, *FBS* fasting blood sugar, *AlkP* alkaline phosphatase, *IU* international unit, *HDL* high-density lipoprotein, *LDL* low-density lipoprotein, *GGT* gamma-glutamyl transferase, *FLI* fatty liver index, *DII* dietary inflammatory index. *P*-value ˂0.05 is considered significant. * indicates significance

As described in Table 1, the mean age of subjects was just under 49 years, the mean fatty liver index (FLI) was 40, and the mean DII was -0.30. NAFLD, defined as FLI ≥ 60, was present in 26.9% of individuals. The rate of opium consumption, smoking, hypertriglyceridemia (HT), and physical activity were significantly higher among men than women, while diabetes mellitus (DM), hypertension, hypercholesterolemia (HC), and NAFLD were more prevalent among women compared to men. Additionally, the mean DII was significantly higher in female subjects (*p *= 0.031).

**Fig. 2:**
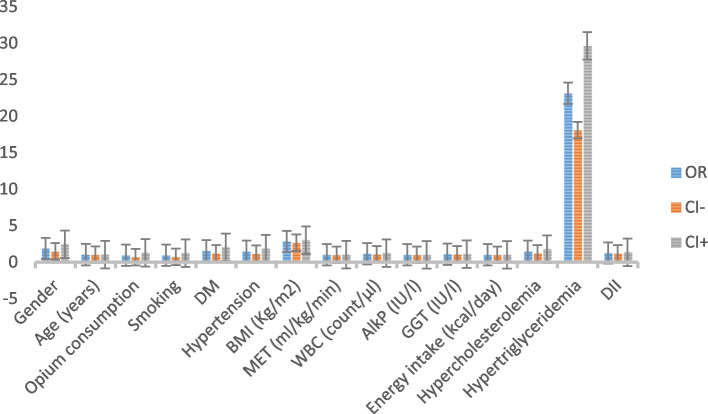
OR and 95% CI of the predictors of NAFLD based on the logistic regression analysis among the 9792 subjects, Fasa PERSIAN cohort study, 2014–2019. (Made by Microsoft Excel)

Results of the adjusted logistic regression analysis shown in Table 2 identified female gender (*p *<0.0001, OR = 1.883), hypertension (*p *= 0.002, OR = 1.463), DM (*p *= 0.001, OR = 1.543), HC (*p *<0.0001, OR = 1.461), HT (*p *<0.0001, OR = 23.140), higher age (*p *<0.0001, OR = 1.027), body mass index (BMI) (*p *<0.0001, OR = 2.834), WBC count (*p *= 0.000, OR = 1.146), and GGT (*p *<0.0001, OR = 1.078) as the independent correlates of NAFLD. As depicted in Fig. 2, HT is the strongest predictor of NAFLD. Additionally, and most importantly, our results indicate that DII can be a valid predictor of NAFLD incidence (*p *<0.0001, OR = 1.254), which supports our hypothesis. Conversely, opium consumption, smoking, physical activity, serum alkaline phosphatase, and daily energy intake were not significant predictors of NAFLD.

**Table 2:** The association between variables and incidence of NAFLD by logistic regression analysis among the 9792 subjects, Fasa PERSIAN cohort study, 2014–2019

	Crude logistic regression			Adjusted logistic regression		
Variables	*P*-value	OR	95% CI	*P*-value	OR	95% CI
Gender	< 0.0001*	0.624	0.569—0.684	< 0.0001*	1.883	1.458 – 2.431
Age	0.003*	1.007	1.002 – 1.012	< 0.0001*	1.027	1.015 – 1.039
DM	< 0.0001*	2.246	1.985 – 2.542	< 0.0001*	1.543	1.185 – 2.010
HTN	< 0.0001*	2.247	2.235 – 2.748	0.002*	1.463	1.156 – 1.851
BMI (Kg/m^2^)	< 0.0001*	1.910	1.857 – 1.964	0.001*	2.834	2.667 – 3.012
MET (ml/kg/min)	< 0.0001*	0.972	0.968 – 0.977	0.58	0.998	0.988 – 1.007
DII	< 0.0001*	2.433	2.342 – 2.528	< 0.0001*	1.254	1.178—1.334
WBC (count/µl)	< 0.0001*	1.216	1.175 – 1.258	0.003	1.146	1.067 – 1.230
AlkP (IU/l)	< 0.0001*	1.003	1.003 – 1.004	0.21	1.001	0.999 – 1.003
GGT (IU/l)	< 0.0001*	1.047	1.043 – 1.051	< 0.0001*	1.078	1.070 – 1.085
Energy intake (kcal/day)	0.001*	1.000	1.000 – 1.000	0.18	1.000	1.000 – 1.000
HC	< 0.0001*	2.361	2.152 – 2.590	< 0.0001*	1.461	1.203 – 1.776
HT	< 0.0001*	6.063	5.496 – 6.688	< 0.0001*	23.140	18.076 – 29.622
Opium consumption	< 0.0001*	0.533	0.473 – 0.601	0.66	0.932	0.684 – 1.270
Smoking	< 0.0001*	0.552	0.494 – 0.617	0.64	0.936	0.707 – 1.239

*DM* diabetes mellitus, *HTN* hypertension, *BMI* body mass index, *MET* metabolic equivalent, *DII* dietary inflammatory index, *WBC* white blood cell, *AlkP* alkaline phosphatase, *GGT* gamma-glutamyl transferase, *HC* hypercholesterolemia, *HT* hypertriglyceridemia, *OR* odds ratio. *indicates significance

Since gender was significantly associated with NAFLD incidence, we carried out the analysis of each gender separately (Table 3). Based on our findings, smoking, and opium consumption were neither associated with NAFLD incidence in women nor men. Again, hypertriglyceridemia was the most potent predictor of NAFLD in both genders (OR = 32.40 in men and OR = 19.02 in women). Most importantly, DII was significantly associated with NAFLD incidence in both genders (OR = 1.36 in men and OR = 1.20 in women).

**Table 3:** The association between variables and incidence of NAFLD by gender by logistic regression analysis among the 9792 subjects, Fasa PERSIAN cohort study, 2014–2019

	Females				Males			
	Crude regression		Adjusted regression		Crude regression		Adjusted regression	
Variables	*P*-value	OR	*P*-value	OR	*P*-value	OR	*P*-value	OR
Age	< 0.0001*	1.013	< 0.0001*	1.040	0.594	0.998	-	-
DM	< 0.0001*	1.964	0.001*	1.722	< 0.0001*	2.476	0.577	1.156
HTN	< 0.0001*	2.301	0.007*	1.486	< 0.0001*	2.298	0.421	1.219
BMI (Kg/m^2^)	< 0.0001*	1.924	< 0.0001*	2.769	< 0.0001*	2.218	< 0.0001*	3.034
MET (ml/kg/min)	< 0.0001*	0.965	0.042*	0.982	< 0.0001*	0.980	0.682	1.002
DII	< 0.0001*	2.357	< 0.0001*	1.205	< 0.0001*	2.532	< 0.0001*	1.360
WBC (count/µl)	< 0.0001*	1.251	0.029*	1.104	< 0.0001*	1.191	< 0.0001*	1.256
AlkP (IU/l)	< 0.0001*	1.004	0.534	1.001	< 0.0001*	1.002	0.934	1.000
GGT (IU/l)	< 0.0001*	1.056	< 0.0001*	1.081	< 0.0001*	1.054	< 0.0001*	1.078
Energy intake (kcal/day)	0.024*	1.000	0.729	1.000	0.543	1.000	-	-
HC	< 0.0001*	2.062	< 0.0001*	1.543	< 0.0001*	2.685	0.126	1.300
HT	< 0.0001*	4.881	< 0.0001*	19.029	< 0.0001*	9.478	< 0.0001*	32.409

*DM* diabetes mellitus, *HTN* hypertension, *BMI* body mass index, *MET* metabolic equivalent, *DII* dietary inflammatory index, *WBC* white blood cell, *AlkP* alkaline phosphatase, *GGT* gamma-glutamyl transferase, *HC* hypercholesterolemia, *HT* hypertriglyceridemia, *OR* odds ratio. *indicates significance

## Discussion

Although previous investigations have assessed the association between dietary patterns and the prevalence of NAFLD, this is the first study directly to evaluate the association between the DII and the incidence of NAFLD in a large cohort population. After adjusting for covariates using logistic regression, we observed that the DII is independently and significantly associated with NAFLD incidence, which confirms our hypothesis.

This finding also aligns with the results of a previous similar case-control study on 295 NAFLD cases and 704 controls in 2019 in Iran, which found that the DII and FLI are correlated [17]. However, our study is the first cohort study on a much larger population in Iran assessing this association. Additionally, several confounding variables that are important in the relationship between DII and NAFLD were missing in the statistical analysis of the previous study. Some of these variables include METS, fasting blood sugar, serum levels of HDL, alkaline phosphatase (Alk-P), and gamma-glutamyl transferase (GGT).

We also showed that the prevalence of NAFLD increases with age, and it is more prevalent among women, as are HC, DM, and hypertension. Our results support the previous findings that the Mediterranean diet, which is rich in whole-grain cereals, vegetables, fruits, and fishes (especially fatty fishes rich in omega-3), is associated with a lower inflammatory index with decreased prevalence of NAFLD. On the other hand, dietary patterns, mostly consisting of foods with a higher inflammatory index, such as western dietary patterns rich in sugar, red meat, processed foods, saturated fats, and soda, are associated with an increased prevalence of NAFLD [28]. A case-control study carried out in 2018 also showed that NAFLD is significantly less common in individuals consuming foods low in fat and cholesterol, in contrast with patients who used fattier foods [11].

Reviewing the possible mechanisms of liver injury induced by foods with high inflammatory potential, a previous animal study showed that feeding rats with high-fat foods resulted in liver damage due to oxidative stress (lipotoxicity) mediated by elevated reactive oxygen species (ROS) production and NADPH oxidase activity (which is regulated by pro-inflammatory cytokines). On the other hand, the antioxidant system was weakened due to a lower level of catalase, glutathione peroxidase (GPX), and superoxide dismutase (SOD) enzymes [29]. It is discussed that the protective effect of the Mediterranean diet against NAFLD incidence and progression is due to the anti-inflammatory and anti-oxidative properties of foods in this regimen [30]. Another finding in our study was that daily energy intake was not associated with the incidence of NAFLD, which is against the existing evidence [31]. However, it should be noted that the energy intake is calculated based on the daily food regimen of individuals, which may be reported inaccurately. Therefore, this finding may be biased.

Another case-control investigation in 2018 concluded that NAFLD is significantly associated with certain "unhealthy" dietary patterns, especially sweet and sugary foods [16]. A possible mechanism proposed for these findings was previous evidence that consuming sugar-rich foods are associated with systemic inflammation and insulin resistance [32, 33]. In an animal study published in 2015, Szabo et al. fed male mice a diet rich in fat, cholesterol, and sugar. They observed that these mice developed pathologic liver changes such as steatohepatitis, liver fibrosis, and tumors. Interestingly, they found that the incidence of steatohepatitis was correlated with serum levels of the inflammatory markers monocyte chemoattractant protein (MCP), TNF-α, and IL-1β, which highlights the association between systemic inflammatory status and fatty liver [16, 34]. The relationship between red meat consumption and NAFLD is also suggested to be due to immune responses and the production of pro-inflammatory cytokines triggered by eating such animal foods [35].

In previous studies, WBC has been found to be independently associated with NAFLD incidence [32, 33], which is also confirmed by our study. Notably, this relationship is independent of metabolic factors [36]. Instead, it is argued that the association is mediated by two related factors: chronic inflammation and elevated oxidative stress. Indeed production of inflammatory cytokines by hepatocytes may contribute to the increased production of WBCs [37].

The role of the immune response in NAFLD has been previously reviewed in detail. In summary, it is claimed that oxidative stress and free fatty acids primarily induce the inflammatory cascades in the liver, leading to the apoptosis of hepatocytes. However, the disease progression is mostly mediated by toll-like receptors, especially in Kupffer cells. High levels of lipopolysaccharides trigger the Kupffer cells leading to inflammatory responses and the production of inflammatory cytokines (i.e., TNF-α and interleukins 6, 10, 12, 18, and 1β). This inflammatory process stimulates liver fibrosis and the disease's progression toward its final stages [38].

Notably, NAFLD itself can trigger inflammatory processes that may lead to serious multi-organ complications, such as cardiovascular diseases and cognitive impairments (e.g., Alzheimer's disease) [39]. Furthermore, there is evidence that the relationship between NAFLD and insulin resistance is bidirectional, meaning that NAFLD also may induce insulin resistance [40, 41].

GGT, a serum marker of liver injury, was found to be significantly associated with NAFLD. This marker represents this disease's histological damage (especially fibrosis) [42, 43]. Our findings also are supported by a previous cross-sectional study suggesting that increased GGT is associated with NAFLD incidence [44].

BMI is another factor significantly associated with NAFLD, which is consistent with a previous cross-sectional study that introduced BMI as a dose-dependent risk factor for developing NAFLD [45]. Additionally, because NAFLD is closely related to metabolic syndrome [46], we entered hypertension and DM type II into our analysis and concluded that both could predict NAFLD incidence. Two recent large-scale meta-analysis studies showed that NAFLD is associated with at least a two-fold increase in DM incidence [47, 48]. Furthermore, DM typre II is associated with increased hepatocellular carcinoma in patients with NAFLD-related cirrhosis [49].

It has been previously discussed that there is a causal relationship between hypertension and NAFLD (hypertension as a risk factor of NAFLD), which components of the metabolic syndrome may mediate (e.g., central obesity, DM type II, and insulin resistance) [50]. However, there is no consensus regarding the causal link between NAFLD and hypertension. A prospective cohort study in 2014 on 22,090 Korean men showed the opposite finding, as NAFLD is a risk factor for hypertension [51]. Although this conflict still persists, the causal association between NAFLD and hypertension might be reciprocal, as both NAFLD and hypertension increase the risk of the other one. Therefore, the diagnosis of NAFLD should be considered in patients with hypertension and vice versa. Additionally, more studies are required to evaluate the cost-effectiveness of referring hypertensive patients for checking liver function tests. It must be considered that even minor rises in liver enzymes should not be ignored in patients with hypertension [50].

Finally, we found both HT and HC to be significantly and positively correlated with an increased risk of NAFLD. A previous cross-sectional study on 268 NAFLD patients also found that HT and HC are associated with an increased risk of hepatic steatosis [52]. In the context of a longitudinal cohort study, Harlow et al. also suggested that every child with NAFLD should undergo lipid profile screening [53]. Besides, Ju et al. claimed that HT is significantly associated with NAFLD, and this association is independent of obesity [54].

Because the diagnosis of DM and hypertension cannot be established based on a single measurement of FBS or blood pressure, we relied on participants' self-report of doctor-diagnosed conditions, which might introduce information biases to our data. Second, the food frequency questionnaire (FFQ) was filled out based on participants' self-report, which might also entail information biases. Likewise, the calculation of daily energy intake may be inaccurate, which may be responsible for the unexpected result of an absence of significant association between the daily energy intake and NAFLD. Additionally, due to cultural taboos, some alcohol drinkers may avoid declaring it. Another important limitation of this study was that we were not able to carry out imaging assessments (e.g., MRI or ultrasound) or liver biopsy to establish the diagnosis of NAFLD for all individuals. However, FLI is still a reliable and relatively precise tool for diagnosing NAFLD [25]. Finally, this study had a cross-sectional design, and therefore, we could not infer causality due to temporal confusion. We suggest that further longitudinal studies with long-term follow-ups are necessary to determine the causal link between NAFLD and other backgrounds, metabolic, and nutritional factors. The strength of this study is that it was carried out with data extracted from a large population which reinforces the precision of our findings. Additionally, the interviews, samplings, and other procedures were conducted by trained individuals, which helped minimize the biases during the data collection.

## Conclusion

Conclusively, according to our results, there is a significant and positive predictive association between DII and the incidence/odds of NAFLD, indicating that having a dietary pattern with higher inflammatory potential, usually consisting of sugary, saturated fatty acids, and processed foods is associated with increased risk of developing NAFLD. We also observed that NAFLD is correlated with DM, hypertension, HC, and HT, highlighting the relationship between NAFLD and metabolic syndrome.

## Data Availability

The datasets used in the present study are not available publicly but are available from the corresponding author at reasonable request.

## Abbreviations

Alk-P: Alkaline phosphatase
BMI: Body mass index
DII: Dietary inflammatory index
FFQ: Food frequency questionnaire
FLI: Fatty liver index
GGT: Gamma glutamyl transferase
GPX: Glutathione peroxidase
HT: Hypertriglyceridemia
HC: Hypercholesterolemia
IL: Interleukin
MCP: Monocyte chemoattractant protein
NADPH: Nicotinamide adenine dinucleotide phosphate
NAFLD: Non-alcoholic fatty liver disease
OR: Odds ratio
ROS: Reactive oxygen species
SD: Standard deviation
SOD: Superoxide dismutase
TG: Triglyceride
TNF: Tumor necrosis factor
WBC: White blood cells
WC: Waist circumference
